# The *MMP2* rs243865 polymorphism increases the risk of prostate cancer: A meta-analysis

**DOI:** 10.18632/oncotarget.18014

**Published:** 2017-05-19

**Authors:** Kun Liu, Shuo Gu, Xuzhong Liu, Qing Sun, Yunyan Wang, Junsong Meng, Zongyuan Xu

**Affiliations:** ^1^ Department of Urology, Huai'an First People's Hospital, Nanjing Medical University, Huai'an, China, 223300

**Keywords:** prostate cancer, matrix metalloproteinase 2, polymorphism, meta-analysis

## Abstract

Prostate cancer is a common cancer in men. However, the association between the rs243865 single-nucleotide polymorphisms in the matrix metalloproteinase 2 gene (*MMP2*) and the risk for prostate cancer is inconclusive. We searched the PubMed, EMBASE, Cochrane Library, and the Chinese CNKI and WANFANG databases for the relevant literature. Data were extracted and pooled results were estimated from odds ratios (OR) with 95% confidence intervals (95% CIs). The quality of included studies was assessed, and publication bias of all included studies was examined. A total five studies involving 1895 patients with prostate cancer and 1918 controls were included. There was a significant association between rs243865 polymorphisms and higher risk of prostate cancer in the co-dominant model, dominant model, and allele model (CC vs. CT+TT, OR: 1.60, 95% CI: 1.22–2.11, *P* = 0.001; CC vs. CT, OR: 1.80, 95% CI: 1.34–2.42, *P* < 0.001; C vs. T, OR: 1.32, 95% CI: 1.05–1.66, *P* = 0.016, respectively). However, there was no significant difference between the co-recessive model and recessive model. Our meta-analysis results suggest that *MMP2* rs243865 polymorphisms are significantly associated with higher risk of prostate cancer.

## INTRODUCTION

Prostate cancer is a major public health problem worldwide; it has a multifactorial and complex etiology. It is the second most common male malignancy in the world and the fourth most common cancer overall, accounting for 11% of male cancers and 9% of cancer-related mortality [1, 2]. While the incidence and mortality rates of prostate cancer vary across different regions, the highest rates have been reported in the developed countries [3]. With regard to the high morbidity and mortality rates, early diagnostic methods of prostate cancer remain important but are insufficient for identifying the disease even with the widespread use of serum prostate-specific antigen (PSA) examination in elderly men [4].

Matrix metalloproteinase 2 (*MMP2*), a member of the *MMP* gene family, encodes the zinc-dependent enzymes capable of cleaving components of the extracellular matrix (ECM) and the molecules involved in signal transduction [5]. Numerous studies have indicated the crucial role of the *MMP2* gene in the pathogenesis of the initiation, invasion, and metastasis of various tumors, such as ovarian cancer, hepatocellular carcinoma, prostate cancer, and lung cancer [6–9]. It has been suggested that the *MMP2* gene is strongly associated with the development of prostate cancer by affecting cell growth, the production of cell junction proteins, such as collagens, and the pathogenesis of metastasis and invasion [10–12]. Furthermore, certain single-nucleotide polymorphism (SNP) mutations of the *MMP2* gene, especially at position −1306 of the *MMP2* promoter (rs243865), function as regulatory factors in the formation and metastasis of prostate cancer [13]. However, the results were conflicting when these studies were taken together.

In this study, we performed a literature review and conducted a meta-analysis of the pooled results of relevant data from studies exploring the association between *MMP2* genetic polymorphisms and the risk of prostate cancer.

## RESULTS

### Study characteristics

**Figure 1:**
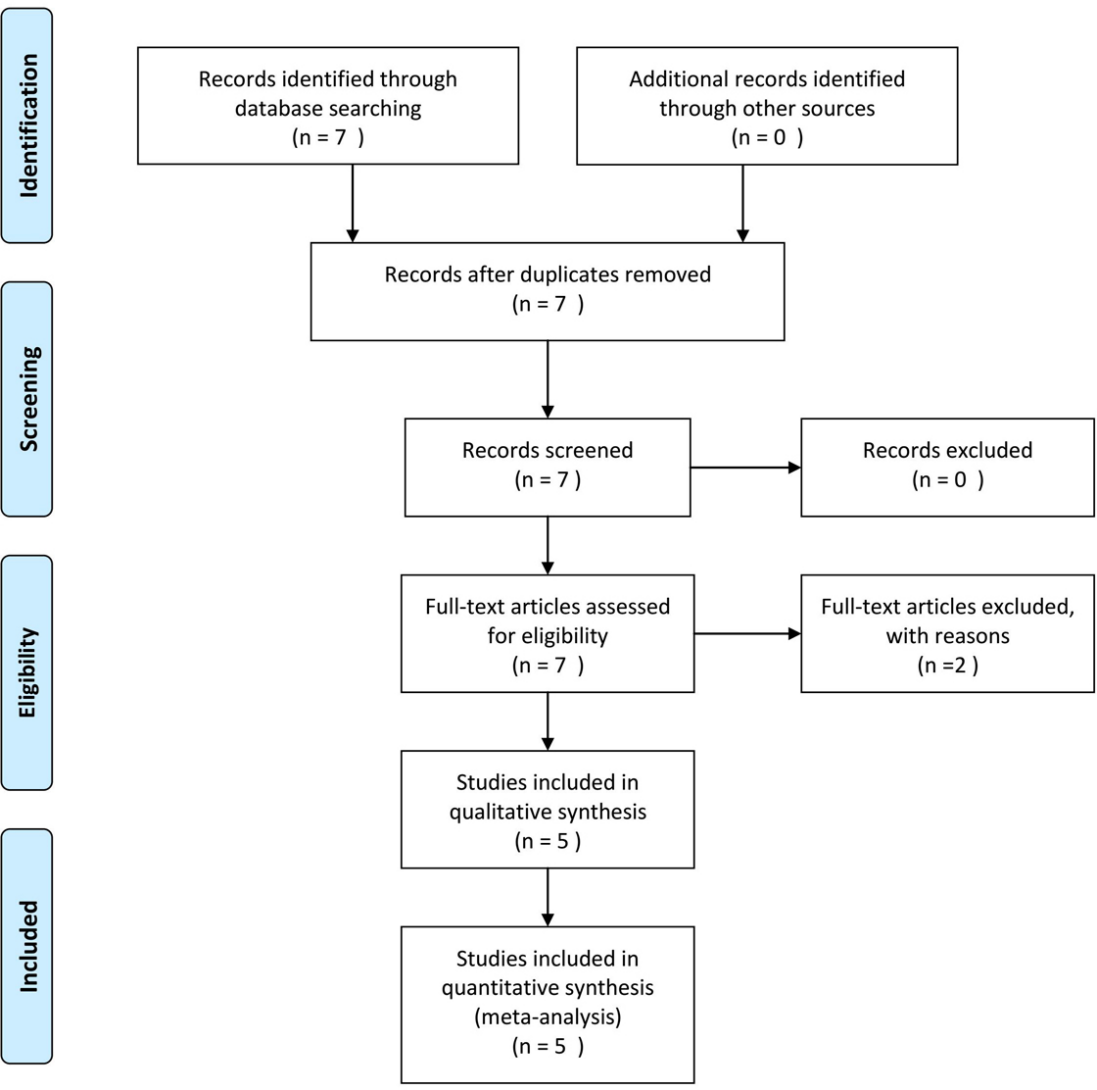
Flow diagram of study selection

Figure 1 shows the flow diagram for the literature search. Based on the search strategy, seven articles were identified in the initial search. After the full-text review, two studies were excluded based on the inclusion and exclusion criteria. Subsequently, a total five studies involving 1895 cases and 1918 controls were included in our meta-analysis [13–17].

Table 1 shows the main characteristics of the included studies. All studies were published in English. The publication year ranged 2008–2016. The ethnicities studied were Asian, Caucasian, and Latino. Only one *MMP2* SNP (rs243865) could be included for meta-analysis. Only one study [14] performed Hardy–Weinberg equilibrium (HWE) testing. The quality of primary studies as assessed by the Newcastle-Ottawa Scale (NOS) showed that except the study by Srivastava et al. [14], which was high-quality, the studies were all medium-quality.

**Table 1:** Main characteristics of eligible studies

Author	Ethnicity	Case number (Con/PC group)	Mean age (years; mean ± SD)	Genotyping methods	HWE test	NOS score
**L S. Salavat (2016)**	Asian	54/50	Con: 60.17 ± 8.7; PC: 65.46 ± 8.99	PCR	NA	6
**Z. Adabi (2015)**	Asian	139/102	NA	PCR	NA	6
**P. Srivastava (2012)**	Asian	200/190	Con: 59.1 ± 10.4; PC: 62.6 ± 8.9	PCR-RFLP	Yes	7
**ST. d Reis (2009)**	Latino	100/100	65 ± 6.9	Taqman	NA	6
**E J. Jacobs (2008)**	Caucasian	1425/1453	NA	MassARRAY system	NA	5

Abbreviations: Con, control; PC, prostate cancer; SD, standard deviation; HWE, Hardy-Weinberg equilibrium; NOS, Newcastle-Ottawa Scale; PCR-RFLP, PCR- Restriction fragment length polymorphism; NA, not available.

### Pooled results of meta-analysis

**Figure 2:**
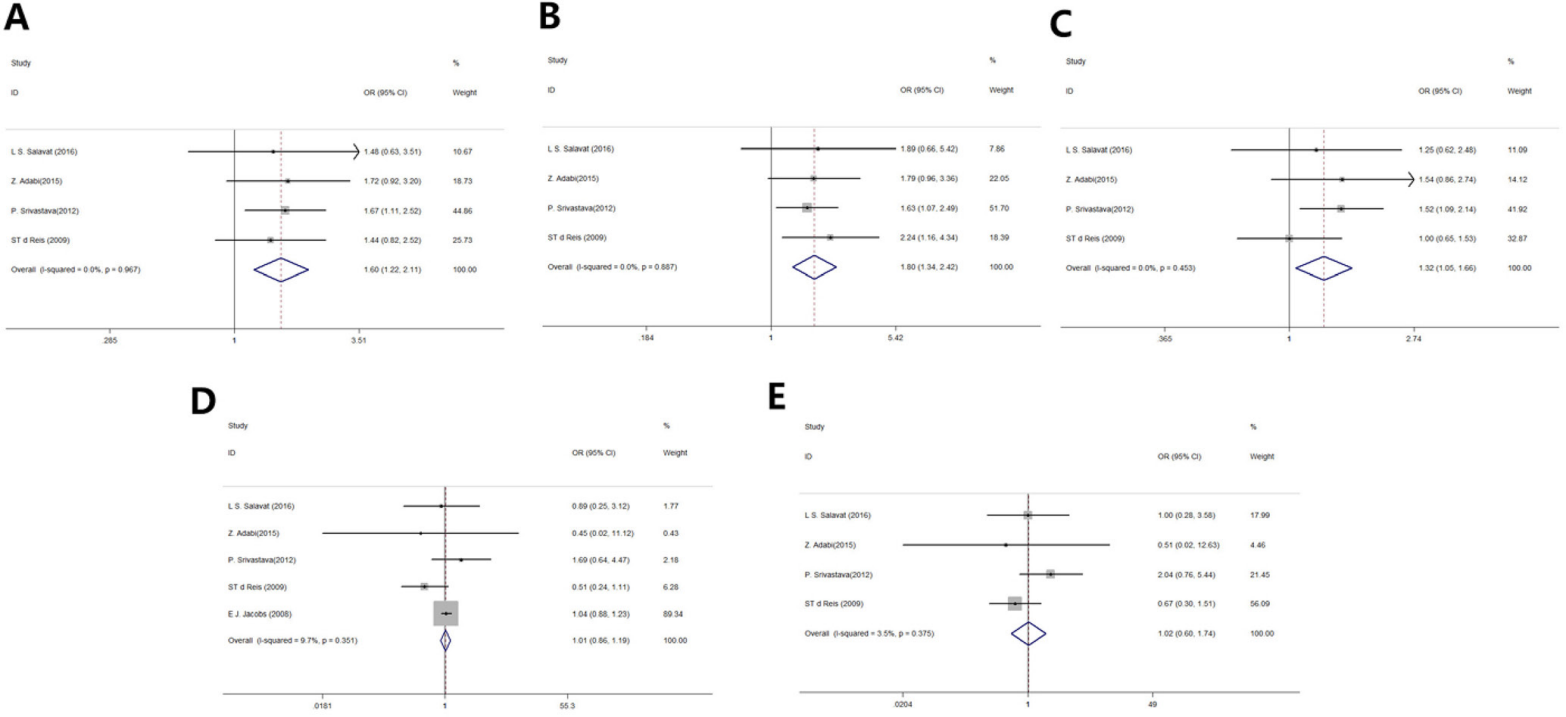
Forest plot of *MMP2* rs243865 polymorphisms and prostate cancer risk (**A**) Co-dominant model. (**B**) Dominant model. (**C**) Allele model. (**D**) Co-recessive model. (**E**) Recessive model.

Table 2 summarizes the results on the association between *MMP2* −1306C/T SNPs and the risk of prostate cancer. Four studies were included in the co-dominant analysis, and there was a significant difference between two groups (CC vs. CT+TT, odds ratio [OR]: 1.60, 95% confidence interval [95% CI]: 1.22–2.11, *P* = 0.001; Figure 2A). Similarly, there was a significant difference between patients with prostate cancer and the controls in the dominant and allele models (CC vs. CT, OR: 1.80, 95% CI: 1.34–2.42, *P* < 0.001; Figure 2B; C vs. T, OR: 1.32, 95% CI: 1.05–1.66, *P* = 0.016; Figure 2C, respectively). No statistical significance was found in the co-recessive and recessive models between two groups (TT vs. CC+CT, OR: 1.01, 95% CI: 0.86, 1.19, *P* = 0.87; Figure 2D; CC vs. TT, OR: 1.02, 95% CI: 0.60, 1.74, *P* = 0.95, respectively; Figure 2E).

**Table 2:** Results of meta-analysis of MMP2 −1306C/T polymorphisms and PC risk

Model	Polymorphisms	Eligible studies	OR	95% CIs	*P* value	*I*^2^	*P* value
**Co-dominant**	CC vs. CT+TT	4	1.60	1.22, 2.11	**0.001**	0.0%	0.97
**Co-recessive**	TT vs. CC+CT	5	1.01	0.86, 1.19	0.87	9.7%	0.35
**Recessive**	CC vs. TT	4	1.02	0.60, 1.74	0.95	3.5%	0.38
**Dominant**	CC vs. CT	4	1.80	1.34, 2.42	**< 0.001**	0.0%	0.89
**Allele**	C vs. T	4	1.32	1.05, 1.66	**0.016**	0.0%	0.45

Abbreviations: MMP2, matrix metalloproteinase 2; PC, prostate cancer; OR, odds ratio; 95% CIs, 95% confidential intervals.

There was no heterogeneity in any genetic model (Table 2). One-way sensitivity analysis was performed to determine the degree to which an individual study affected the overall OR estimates, and showed that excluding individual studies did not affect the pooled ORs and 95% CIs. Furthermore, sensitivity analysis revealed no significant publication bias in any genetic model (*P* > 0.05).

## DISCUSSION

Prostate cancer is one of the most common cancers in men. MMP2 is involved in tumor pathogenesis, which plays a key role in tumor cell invasion and metastasis. Thus, genetic variants that influence the level of *MMP2* gene expression or protein function could contribute to tumor invasion and metastasis [18]. In our meta-analysis, it is indicated for the first time that the *MMP2* rs243865 SNP is significantly correlated with the risk of prostate cancer.

MMPs are zinc metalloproteases that degrade ECM collagens, which are important in tissue remodeling and repair during development and inflammation [19]. Among the MMP genes, gelatinase A, encoded by the *MMP2* gene, has been specifically correlated to tumor pathogenesis [20, 21]. Furthermore, the *MMP2* promoter contains several cis-acting regulatory elements, which modulate *MMP2* expression through transcription factors, such as p53 and Sp1 [22, 23]. Among these *MMP2* promoter SNPs, C/T transition at nucleotide −1306 disrupts the Sp1-binding position at the T allele (CCACC BOX) and has significantly lower transcription activity when compared to the C allele [24]. In the present study, the co-dominant model, dominant model, and allele model results suggest that subjects carrying the T allele have higher prostate cancer risk compared to those with the C allele, which could be because T alternation in rs243865 could decrease promoter activity in two different luciferase reporter gene constructs: one in the context of the Sp1 regulatory element and the other in the background of the native *MMP2* promoter [25]. Sp1 is a ubiquitously expressed transcription factor that regulates a variety of genes in a constitutive or inducible manner and exerts a synergistic effect essential for modulating gene activation [26, 27]. Recently, a meta-analysis that investigated the association of the *MMP2* −1306C/T polymorphism with cancer risk reported that the SNP was significantly correlated with reduced risk of cancer, which is consistent with our results [25]. Taken together, the *MMP2* −1306C/T SNP is significantly associated with reduced risk of prostate cancer, and could be a candidate SNP for prostate cancer diagnosis.

Nevertheless, our findings should be interpreted with caution, given the limitations of this study. First, only studies published in English and Chinese were included. Next, limited by the number of eligible studies, we could not perform subgroup analysis of prostate cancer based on the Gleason score or tumor-node-metastasis (TNM) classification. Therefore, the statistical power might be insufficient for assessing the relationship in prostate cancer.

In summary, we show that the *MMP2* −1306C/T polymorphism is a susceptibility locus for prostate cancer. Individuals carrying the T allele are significantly associated with increased prostate cancer risk. Large-scale, well-designed studies should be conducted to confirm our results and explore the mechanisms further.

## MATERIALS AND METHODS

### Search strategy

A comprehensive literature search was performed using the PubMed, Cochrane Library Central Register of Controlled Trials (CENTRAL), EMBASE, and the Chinese CNKI and WANFANG databases (updated April 1, 2017) by two authors (K L and XZ L) independently. The following keywords were used: (matrix metalloproteinase OR MMP2), AND (polymorphisms OR SNPs OR variants), AND (MESH item, prostatic neoplasms). The equivalent Chinese terms were used in the Chinese databases. Furthermore, the reference lists of all studies included in the meta-analysis were reviewed for possible inclusion.

### Inclusion and exclusion criteria

The inclusion criteria were: (1) case–control studies designed to investigate the relationship between *MMP2* SNPs and prostate cancer risk; (2) available information on the genotype or allele frequencies in case and control groups; (3) all subjects from three allelic groups were from a population within the same geographic area and ethnic background; (4) full-text article published in English or Chinese. Studies with insufficient data for pooling and with no genotype frequencies for each polymorphism and outcome were excluded. Reviews or studies based on non-human research were also excluded. Two authors (K L and XZ L) independently assessed and selected studies for final analysis; discrepancies were resolved by consensus.

### Data extraction and quality assessment

Two investigators (K L and XZ L) independently reviewed and extracted the relevant data from all included studies and reached consensus for all items. The following data were extracted from each included study: (1) first author's name and year of publication, (2) ethnicity, (3) number of controls and cases, (4) sex and mean age of controls and cases, (5) genotyping method and HWE of the controls, (6) allele or genotype frequencies of cases and controls. Missing data were examined by contacting the first or corresponding author. The quality of each included study was assessed according to a methodological quality assessment scale that had been extracted and modified from previous studies [28]. A total score of ≤ 3, 4–6, and ≥ 7 indicated low, medium, and high quality, respectively.

### Statistical analysis

The pooled data were used to assess the strength of the association between *MMP2* polymorphisms and prostate cancer risk using OR with 95% CIs in dominant, recessive, co-dominant, co-recessive, and allele models. *P* < 0.05 was considered statistically significant. Heterogeneity among studies was determined by *I^2^* and was defined as 100% × (Q - df)/Q, where Q is Cochran's heterogeneity statistic and df is the degrees of freedom, with a fixed-effect model selected at low statistical inconsistency (*I^2^* < 25%). Otherwise, a random-effect model, which is better adapted to clinical and statistical variations, was selected [29]. To explore the potential effects of heterogeneity, stratification analysis by ethnicity, age, and quality criteria was carried out. Egger's regression test and funnel plots were used to assess potential publication bias. Cumulative meta-analysis was carried out based on the year of publication. All analyses were performed using STATA (release 12.0, College Station, TX, USA).
